# Lack of Association between Mannose-binding Lectin 2 Codons 54 and 57 Gene Polymorphisms and Cervicovaginal Infections in Mexican Women

**Published:** 2017-06

**Authors:** Nadia Velazquez-Hernandez, Marisela Aguilar-Duran, Alma Rosa Perez-Alamos, Sergio Estrada-Martinez, Jose M. Salas-Pacheco, Luis Francisco Sanchez-Anguiano, Ada. A. Sandoval-Carrillo, Brissia Lazalde-Medina, Cosme Alvarado-Esquivel

**Affiliations:** 1 Instituto de Investigacion Cientifica, Universidad Juarez del Estado de Durango. Avenida Universidad S/N. 34000 Durango, Mexico;; 2 Facultad de Medicina y Nutrición, Universidad Juarez del Estado de Durango. Avenida Universidad S/N. 34000 Durango, Mexico;; 3 Unidad de Investigacion Biomedica Instituto Mexicano del Seguro Social, Durango, Durango. Predio Canoas 100. 34067 Durango, Mexico

**Keywords:** Cervicovaginal infections, MBL2 gene, codon 54, codon 57

## Abstract

The mannose-binding lectin (MBL) 2 gene has an important function in the innate immune response and activation of the third pathway of the complement system. Some studies have assessed the association of the MBL2 gene polymorphisms with cervicovaginal infections (CVI); however, there is no information about this association in Mexican women. This study aimed to determine the association between the MBL2 codons 54 and 57 gene polymorphisms with CVI in a sample of Mexican women. Through a cross-sectional study, blood samples and cervicovaginal cultures were obtain from 354 women. MBL2 genotyping was performed by real-time polymerase chain reaction with Taqman probes. Of the 354 women studied, 128 (36.2%) had CVI and 226 (63.8%) were healthy. The frequencies of the C and T variants in codon 54 in women with CVI were 83% and 17%, respectively; whereas the frequencies of these variants in healthy women were 82% and 18%, respectively. The frequencies of variants C/C, C/T, and T/T in women with CVI were 68%, 31%, and 1%, respectively; whereas the frequencies of these variants in healthy women were 68%, 29%, and 3%, respectively. With respect to codon 57, the frequencies of variants C and T were identical in women with CVI and in healthy women (97% and 3%, respectively). The frequencies of variants C/C, C/T, and T/T were identical in women with CVI and in healthy women (94%, 6%, and 0%, respectively). We conclude that MBL2 codons 54 and 57 gene polymorphisms do not associate with CVI in Mexican women.

## INTRODUCTION

Cervicovaginal infections (CVI) are a group of gynecological entities characterized by replacement of normal vaginal flora with infectious agents including virus, bacteria, fungi and protozoa (1, 2). CVI occur in women of any age and are one of the most important causes of medical consultations in primary healthcare centers (3, 4). It is estimated that 90% of CVI are caused by three groups of pathogens: a) anaerobic bacteria, mainly *Gardnerella vaginalis* leading to bacterial vaginosis; b) yeasts of the *Candida spp* genus leading to vulvovaginal candidiasis; and c) the protozoan parasite *Trichomonas vaginalis* (5-7). The innate immune system represents the first line of defense against infectious agents leading to an immediate response through several effector mechanisms that recognize and remove pathogens, and activate the adaptive immune system (18, 9). The mannose-binding lectin (*MBL*) 2 is a protein codified by a gene located on chromosome 10q11.2 (10, 11). This serum lectin is synthetized by the liver and it is released to the blood stream during the innate immune response against virus, bacteria, yeasts, and parasites (12). The *MBL* binds cell surface carbohydrates of pathogens mediating opsonization either directly or through complement activation by the lectin pathway (13). Low or deficient concentrations of MBL in serum are mainly due to single nucleotide polymorphisms of exon 1 of MBL2 gene (14). MBL2 codons 54 and 57 gene polymorphisms (variant allele O; wild-type allele designated as A) are denoted as B and C, respectively (15). These point mutations result in amino acid substitutions in the collagen region: in codon 54 (GGC->GAC, Gly->Asp, allele B), and in codon 57 (GGA->GAA, Gly->Glu, allele C) (16-18). Several studies have reported the association of MBL2 polymorphism with CVI (19-21). However, there is not any report about this association in Mexican population.

## MATERIALS AND METHODS

### Selection and description of participants

Through a cross-sectional study, 354 women attending consultations in the Family Healthcare Department in the Institute for Scientific Research of the Juárez University of Durango State in Durango City, Mexico were examined. Women were enrolled in the study from November 2014 to June 2016. Inclusion criteria for enrollment were: 1) age 17 years and older; 2) sexually active; and 3) who voluntarily accepted to participate in the study. Exclusion criteria were: 1) pregnant women, 2) women during menstrual period; 3) women under treatment for CVI in the last 10 days (including vaginal ovules, creams, or douching); 4) women with a recent miscarriage or postpartum; 5) history of hysterectomy; 6) suffering from autoimmune or systemic diseases; and 7) under treatment with immunosuppressive agents or antibiotics.

### Technical information

Cervicovaginal secretions from participants were obtained with sterile cotton swaps and placed into routine culture media (chocolate agar, blood agar, and Thayer-Martin agar). In addition, a direct microscopic examination of the cervicovaginal secretions for the presence of pathogens was performed. Cervicovaginal secretions were also tested for the presence of amines, and examined using the Gram stain.

DNA was obtained from whole blood of participants by the QIAamp DNA Blood Mini Kit (QIAGEN) following the instructions of the manufacturer. The yield DNA concentration and purity were measured by the NanoDrop 2000 spectrophotometer (Thermo Fisher Scientific Inc., Germering, Germany). Genotyping was performed using a real-time PCR equipment (StepOne, Applied Biosystems, Carlsbad, CA, USA) with TaqMan probes (codon 54: rs1800450; codon 57: rs1800451; Life Technologies, Australia). Typical reactions to a final volume of 20 µl consisted of 10 ng of genomic DNA, 0.625 µl TaqMan SNP genotyping assay, and 5.0 µl of genotyping master mix. Amplification was performed at 60°C for 30 seconds and 95°C for 10 minutes followed by 40 cycles of 92°C for 15 seconds and 60°C for 1 minute, and a final step of 60°C for 30 seconds.

### Ethics aspects

This project was approved by the Ethics Committee of the Institute for Scientific Research of the Juárez University of Durango State, Mexico. An informed consent was obtained from all participants.

### Statistics

Statistical analysis was performed using the software SPSS version.15.0. Allelic, genotypic and haplotypic frequencies were calculated with the aid of the software SNPStats, and odds ratio (OR) and 95% confidence interval (CI) were calculated. *P* values less than 0.05 were considered statistically significant.

## RESULTS

One hundred and twenty-eight (36.2%) of the 354 women studied had CVI. Of them, 72 (20.3%) had bacterial vaginosis, 49 (13.8%) vulvovaginal candidiasis, and 7 (2.0%) trichomoniasis. Two hundred and twenty-six (63.8%) women were healthy. Mean age of women was 36.4 ± 10.3 (range: 17-67) years. Mean age at first sexual relation was 19.3 ± 3.8 (range: 11-40) years. Mean number of sexual partners was 3.0 ± 4.2 (range: 1-50). Mean number of sexual intercourses a month was 6.9 ± 5.6 (range: 0-40), and the median number of miscarriages was 0 (range 0-7). The frequencies of the C and T variants in codon 54 in women with CVI were 83% and 17%, respectively; whereas the frequencies of these variants in healthy women were 82% and 18%, respectively. The frequencies of variants C/C, C/T, and T/T in women with CVI were 68%, 31%, and 1%, respectively; whereas the frequencies of these variants in healthy women were 68%, 29%, and 3%, respectively. No association between the C/C reference genotype, C/T genotype polymorphism (OR=0.98; 95% CI: 0.60-1.58), and T/T genotype (codominant, OR=4.65; 0.55-39.1) and CVI was found. Codon 54 polymorphisms had genotypic distributions consistent with Hardy-Weinberg equilibrium in women with CVI (*P*=0.19), and in healthy women (*P*=1.0). With respect to codon 57, the frequencies of variants C and T were identical in women with CVI and in healthy women (97% and 3%, respectively). The frequencies of variants C/C, C/T, and T/T were identical in women with CVI and in healthy women (94%, 6%, and 0%, respectively). No association between this polymorphism (C/C reference genotype, C/T genotype, OR=1.18; 95% CI: 0.45-3.09) and CVI was found. Codon 57 polymorphisms had genotypic distributions consistent with Hardy-Weinberg equilibrium in women with CVI (*P*=1.0), and in healthy women (*P*=1.0). A correlation of the allelic and genotypic frequencies and the clinical characteristics of the women studied is shown in Table 1. Results of the association analysis of codons 54 and 57 MBL2 haplotypes with CVI are shown in Table 2. No haplotypic association with CVI was found (*P*=0.74).

**Table 1:** Allelic and genotypic frequencies of codons 54 and 57 polymorphisms of MBL2 gene in the women studied

Diagnosis^a^	Codon	Genotype	Positive No. (%)	Allele	Positive No. (%)	*P* b value

Vulvovaginal candidiasis (n=49)	MBL54	C, C	36 (73.5)	C	85(86.7)	
		C, T	13(26.5)			
		T, T	0	T	13(13.3)	0.27
Bacterial vaginosis (n=72)	MBL54	C, C	47(65.3)	C	118(81.9)	
		C, T	24(33.3)			
		T, T	1(1.4)	T	26(18.1)	0.95
Trichomoniasis (n=7)	MBL54	C, C	5 (71.4)	C	12 (85.7)	
		C, T	2 (28.6)			
		T, T	0	T	2 (16.6)	1.00
Controls (n=230)	MBL54	C, C	155(67.4)	C	378(82.1)	
		C, T	68(29.6)			
		T, T	7(3.0)	T	82(17.8)	
Vulvovaginal candidiasis (n=49)	MBL57	C, C	47(95.9)	C	96(97.9)	
		C, T	2(4.1)			
		T, T	0	T	2(2.0)	1.00
Bacterial vaginosis (n=72)	MBL57	C, C	67(93.1)	C	139(96.5)	
		C, T	5(6.9)			
		T, T	0	T	5(3.4)	0.77
Trichomoniasis (n=7)	MBL57	C, C	7 (100.0)	C	14(100.0)	
		C, T	0			
		T, T	0	T	0	1.00
Controls (n=230)	MBL57	C, C	217(94.3)	C	447(97.1)	
		C, T	13(5.7)			
		T, T	0	T	13(2.8)	

a Four women had more than one infection;

b Compared to controls (Fisher exact test).

**Table 2:** Association between codons 54 and 57 haplotypes of MBL2 gene and cervicovaginal infections

Codon 54	Codon 57	Women with cervicovaginal infections (n=124)^a^	Controls (n=230)	OR (95% CI)^b^	*P* value

C	C	81	79.8	-----	----
T	C	16.2	17.4	1.16 (0.73-1.83)	0.53
C	T	2.8	2.5	1.29 (0.47-3.53)	0.62
T	T	0	0.3	c	

General haplotypic association: *P*=0.74.

a Cervicovaginal infections: bacterial vaginosis, vulvovaginal candidiasis, and trichomoniasis;

b Age-adjusted;

c Undefined because of cells with a 0 value.

## DISCUSSION

Cervicovaginal infections represent a public health problem in Mexican women. These infections have a high morbidity, and their complications are important cause of mortality in women at reproductive age (22). The present study aimed to identify the possible influence of codons 54 and 57 polymorphisms of *MBL2* gene on the presence of CVI including bacterial vaginosis, vulvovaginal candidiasis, and trichomoniasis in Mexican women.

Genetic components, vaginal microbiota, and local immunity play not only an important role in the health of women but also may contribute to a higher susceptibility to certain infections (23, 24). MBL2 is a vaginal component that protects against repeated proliferation of atypical vaginal microflora (25). Polymorphisms of the structural region of MBL2 gene, especially codon 54 and in a minor extent codon 57, cause alterations in the mannose-binding lectin production (11). Several studies in women populations have found a significant association between recurrent vaginal infections, especially vulvovaginal candidiasis and bacterial vaginosis, with the presence of codon 54 polymorphisms of MBL2 gene (7, 9, 14, 20, 21, 26). However, this association was not found in our study. It is important to mention that recurrent vulvovaginal infections were not considered in the present study. Only acute infections were included in our study. With respect to codon 54, the genotypic frequencies of homozygotes and heterozygotes variants in our study were 73.5% and 26.5%, respectively. These frequencies differ slightly from other frequencies reported in women with vulvovaginal candidiasis. For instance, frequencies of homozygotes and heterozygotes variants in women in China were 66.6% and 33.3%, respectively (20); whereas, these frequencies in women in Brazil were 64.3% and 35.7%, respectively (9). No mutated homozygotes were found in our study nor in the Chinese and Brazilian studies. Very little is known about the association of MBL2 with parasitic infections (12, 27). In the present study, we examined the association of codons 54 and 57 polymorphisms in women with trichomoniasis; however, this infection was present in only 8 individuals and no association of this infection with the polymorphisms was found. The fact that allele B (T) was not associated with any case of cervicovaginal infections suggests that multiple factors other than local factors can be involved in the pathogenesis of vaginal infections.

No statistically significant difference in the frequencies of the codon 54 variants C/C, C/T, and T/T between women with CVI and healthy women was found (68%, 31%, and 1%; and 68%, 29%, and 3%, respectively). Types of CVI were not associated with codon 54 polymorphisms.

With respect to codon 57 polymorphisms, we did not find mutated homozygotes, only seven women with CVI were heterozygotes: two with vulvovaginal candidiasis and five with bacterial vaginosis. Of the healthy women, 13 were heterozygotes, and there was not association between this polymorphism and CVI. This finding is consistent with others reported in the literature (9).

In the present study, all four possible haplotypes were found. However, no association between these haplotypes and CVI was found. Linkage disequilibrium between codon 54 and codon 57 was detected (D’=0.9903, *P*=0.04). To the best of our knowledge, there are no reports in the literature about the link of these haplotypes with acute CVI. MBL2 polymorphisms have been associated with susceptibility to tubal factor infertility (28).

One limitation of the present study was that we did not measure the concentrations of MBL2 protein in vagina. Further studies to determine the association of MBL2 protein concentrations and codons 54 and 57 polymorphisms in Mexican women are needed.

We conclude that MBL2 codons 54 and 57 gene polymorphisms do not associate with CVI in Mexican women.

## ABBREVIATIONS

CI: Confidence interval
CVI: Cervicovaginal infections
MBL: Mannose-binding lectin
OR: Odds ratio

## References

[1] Gonzalez-Pedraza Aviles A, Ortiz-Zaragoza C, Davila-Mendoza R, Valencia-Gomez CM (2007). Infecciones cervicovaginales más frecuentes; prevalencia y factores de riesgo. Rev. Cubana Obstet Ginecol.

[2] Underhill DM, Iliev ID (2014). The mycobiota: interactions between commensal fungi and the host immune system. Nature Reviews Immunology.

[3] Gonzalez-Pedraza AA, Mota VR, Ortiz ZC, Ponce RR (2004). Factores de riesgo asociados a vaginosis bacteriana. Aten Primaria.

[4] Di Bartolomeo S, Rodríguez M, Sauka D, De Torres R (2002). Prevalencia de microorganismos asociados a secreción genital femenina. Argentina. Revista de Saúde Publica.

[5] Salas Natalia, Ramirez JF, Ruiz B (2009). Prevalencia de microorganismos asociados a infecciones vaginales en 230 mujeres gestantes y no gestantes sintomaticas del Centro de Salud La Milagrosa en el municipio de Armenia (Colombia). Revista Colombiana de Obstetricia y Ginecologia.

[6] Flores-Paz R, Rivera-Sanchez R, Garcia-Jimenez E, Arriaga-Alba M (2003). Etiología de la infeccion cervico vaginal en pacientes del Hospital Juárez de Mexico. Salud Publica de Mexico.

[7] Milanese M, Segat L, De Seta F (2008). MBL2 genetic screening in patients with recurrent vaginal infections. Am. J. Reprod. Immunol.

[8] Merlin-Linares JC, Arce-Hernandez AA, Leyva-Rodríguez A (2006). Lectina de union a manosa: actividad biologica y significado. Rev Cubana Hematol Inmunol Med Transf.

[9] Giraldo PC, Babula O, Gonçalves AK (2007). Mannose-binding lectin gene polymorphism, vulvovaginal candidiasis, and bacterial vaginosis. Obstet. Gynecol.

[10] Thiel S, Frederiksen PD, Jensenius JC (2006). Clinical Manifestations of mannan-binding lectin deficiency. Molecular Immunology.

[11] Villarreal-Alarcon MA, Ortiz-Lopez R, Rojas-Martinez A (2008). Lectina fijadora de manosa en la respuesta inmunitaria innata. Medicina Universitaria.

[12] Padilla-Docal B, Dorta-Contreras AJ, Bu-Coifiu-Fanego R, Callol-Barroso J (2009). Rol de la lectina de union a manosa en infecciones parasitarias. Rev Panam Infectol.

[13] Berron Perez R, Penagos Paniagua MJ, Zaragoza Benitez JM, Rodríguez Alvarez J (2003). El sistema del complemento. Vías clásica y de la lectina que se une a la manosa, Alergia. Asma e Inmunologia Pediatricas.

[14] Babula O, Lazdane G, Kroica J (2003). Relation between Recurrent Vulvovaginal Candidiasis, Vaginal Concentrations of Manoose. Binding Lectin, and a Mannose-Binding Lectin Gene Polymorphism in Latvian Women. Clinical Infections Disease.

[15] Dommett RM, Klein N, Turner MW (2006). Mannose-binding lectin in innate immunity: past, present and future. Tissue Antigens.

[16] Sumiya M, Super M, Tabona P, Levinsky RJ (1991). Molecular basis of opsonic defect in immunodeficient children. Lancet.

[17] Lipscombe RJ, Sumiya M, Hill AV (1992). High frequencies in African and non-African populations of independent mutations in the mannose binding protein gene. Hum. Mol. Genet.

[18] Madsen HO, Garred P, Kurtzhals JA (1994). A new frequent allele is the missing link in the structural polymorphism of the human mannan-binding protein. Immunogenetics.

[19] Donders GGG, Babula O, Bellen G (2008). Mannose-binding lectin gene polymorphism and resistance to therapy in women with recurrent vulvovaginal candidiasis. BJOG.

[20] Liu Fei (2005). Mannose-binding lectin and vulvovaginal candidiasis. International Journal of Gynecology and Obstetrics.

[21] Nedovic B, Posteraro B, Leoncini E, Ruggeri A (2014). Mannose-Binding Lectin Codon 54 GenePolymorphism and Vulvovaginal Candidiasis: A Systematic Review and Meta-Analysis. BioMed Research International.

[22] (2005). Infecciones de transmision sexual y otras infecciones del tracto Reproductivo. Una guia para la practica basica. Organizacion Mundial de la Salud.

[23] Ramirez M, Carlos Dario (2007). Bases geneticas de la susceptibilidad a enfermedades infecciosas humanas. Revista del Instituto Nacional de Higiene Rafael Rangel.

[24] Martin R, Soberon N, Vazquez F, Suarez JE (2008). La microbiota vaginal: composición, papel protector, patología asociada y perspectivas terapéuticas. Enferm Infecc Microbiol Clin.

[25] Bulla R, De Seta F, Radillo O (2010). Mannose-binding lectin is produced by vaginal epithelial cells and its level in the vaginal fluid is influenced by progesterone. Molecular Immunology.

[26] De Seta F, Maso G, Piccoli M (2007). The role of mannose-binding lectin gene polymorphisms in women with recurrent bacterial vaginosis. Am. J. Obstet. Gynecol.

[27] Worthley DL, Bardy PG, Mullighan CG (2005). Mannose‐binding lectin: biology and clinical implications. Internal Medicine Journal.

[28] Laisk T, Peters M, Saare M (2012). Association of CCR5, TLR2, TLR4 and MBL2 genetic variations with genital tract infections and tubal factor infertility. J. Reprod. Imm.

